# Delayed Contrast Medium Excretion Due to Renal Failure After an Emergency Mechanical Thrombectomy for Acute Cerebral Infarction

**DOI:** 10.7759/cureus.74466

**Published:** 2024-11-25

**Authors:** Junpei Nagasawa, Tatuhiro Yokoyama, Eiko Fujimoto, Masamichi Hozumi, Osamu Kano

**Affiliations:** 1 Neurology, Toho University Faculty of Medicine, Tokyo, JPN

**Keywords:** acute cerebral infarction, chronic renal failure, contrast leakage, dual-energy ct (dect), mechanical thrombectomy (mt)

## Abstract

We report a case in which mechanical thrombectomy (MT) was performed on a patient with cerebral infarction and renal failure, and contrast leakage remained on postoperative head computed tomography (CT) scans for more than 24 hours. A 75-year-old woman with a medical history of chronic renal failure due to diabetic nephropathy was admitted to the cardiology department of our hospital with chronic heart failure. During hospitalization, her diabetic nephropathy worsened. Therefore, dialysis was scheduled for introduction, but two days before the scheduled start of dialysis, she suffered a cerebral infarction due to right middle cerebral artery occlusion. Emergency MT was performed, and successful recanalization was achieved. A postoperative head CT scan revealed high-density areas along the cortex in the right frontal and temporal lobes, and this finding persisted for more than three days after the operation. Based on the neurological findings, head magnetic resonance imaging (MRI), and dual-energy CT (DECT) results, this high-density area was diagnosed as contrast agent leakage. The purpose of the current case report is to present a rare case of delayed contrast medium excretion after thrombectomy, management of such cases, and a review of existing literature.

## Introduction

Following mechanical thrombectomy (MT) in patients with acute cerebral infarction, head CT scans often reveal high-density areas [1]. The cerebral hemorrhage and contrast leakage are differentiated. The distinction between these two is often made based on the CT value and the time it takes to disappear. So the contrast leakage generally disappears within 24 hours, but cerebral hemorrhage often persists for more than 24 hours [2]. Here, we report a case in which a mechanical thrombectomy was performed on a patient with cerebral infarction and renal failure, and contrast leakage diagnosed by dual-energy CT (DECT) remained on the image for more than 24 hours.

## Case presentation

A 75-year-old woman with a medical history of hypertension, dyslipidemia, hyperuricemia, diabetes, and chronic renal failure (Kidney Disease Improving Global Outcomes (KDIGO) stage 3) due to diabetic nephropathy (estimated glomerular filtration rate (eGFR)=12.4 ml/min/1.73 m^2^) was admitted to the cardiology department of our hospital with chronic heart failure. During hospitalization, her diabetic nephropathy worsened (eGFR=7.9 ml/min/1.73 m²). Therefore, dialysis was scheduled for introduction. Two days before the scheduled start of dialysis, at 9 a.m., the patient suddenly experienced loss of consciousness and paralysis of the left upper and lower limbs. Examination findings were as follows: blood pressure, 160/86 mmHg; heart rate, 90 beats/min; respiratory rate, 16 beats/min; saturation of peripheral oxygen (SpO2) 99%; and Glasgow Coma Scale (GCS) grade E3V4M6. She was diagnosed with left hemiplegia and right conjugate deviation. The National Institutes of Health Stroke Scale (NIHSS) score was 23. 

The patient’s laboratory test results are shown in Table 1.

**Table 1:** Laboratory test results CRP: C-reactive protein; BUN: blood urea nitrogen; LDL: low-density lipoprotein; HDL: high-density lipoprotein; HbA1c: glycated hemoglobin; PT: prothrombin time; INR: international normalized ratio; aPTT: activated partial thromboplastin time; BNP: brain natriuretic peptide

Investigations	Value
White blood cells	10.4 × 103/μl
Red blood cells	3.82 × 106/μl
Hemoglobin	12.1 g/dl
Platelet	165× 103/μl
CRP	1.5 mg/dl
Na	145 mmol/l
K	3.4 mmol/l
BUN	39 mg/dl
Creatinine	4.43 mg/dl
Triglyceride	77 mg/dl
LDL-cholesterol	43 mg/dl
HDL-cholesterol	68 mg/dl
HbA1c	7.0 %
PT INR	1.0
aPTT	34.9 sec
D-dimer	1.4 μg/ml
BNP	428.6 pg/ml

**Figure 1:**
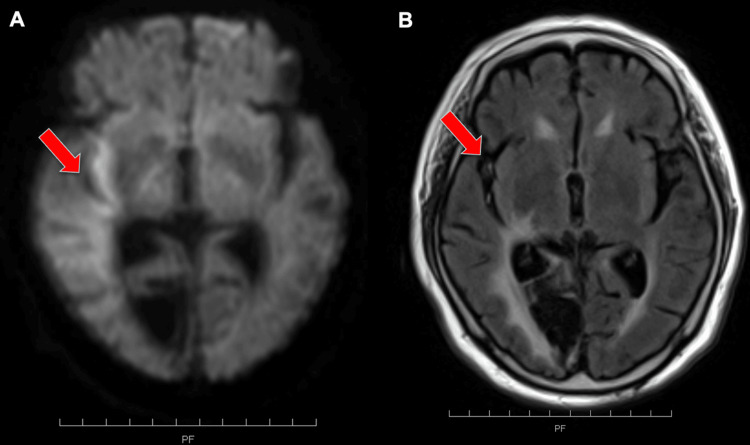
MRI brain of cerebral infarction Diffusion-weighted magnetic resonance imaging (DWI) revealed an acute infarct in the right MCA inferior trunk region (A: arrow), although the image was unclear because of motion artifacts. FLAIR images showed an intra-arterial sign in the middle cerebral artery (MCA) inferior trunk region (B: arrow). FLAIR: fluid attenuated inversion recovery

Magnetic resonance imaging (MRI) of the brain revealed an acute infarct in the right middle cerebral artery (MCA) in the inferior trunk region, although the image was unclear because of motion artifacts (Figure 1A). Fluid-attenuated inversion recovery (FLAIR) images showed a high signal in the blood vessels of the MCA inferior trunk region (intra-arterial sign) (Figure 1B). More than four and a half hours had already passed, so it was determined that a recombinant tissue-type plasminogen activator was not appropriate.

**Figure 2:**
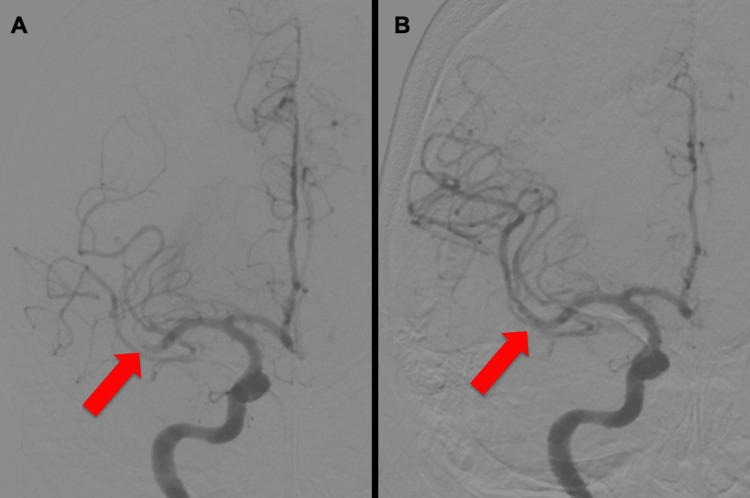
Angiography during mechanical thrombectomy Cerebral angiography revealed occlusion of the right MCA inferior trunk (A: arrow). After mechanical thrombectomy, recanalization of the MCA (B: arrow, TICI grade 3) was confirmed. TICI: thrombolysis in cerebral infarction; MCA: middle cerebral artery

Cerebral angiography revealed occlusion of the inferior trunk of the right MCA (Figure 2A). Based on these findings, emergency MT was indicated. Although the patient had renal failure, she had decided to undergo dialysis; therefore, we concluded that the use of a contrast agent was acceptable. A Trevo NXT 4 × 28 mm (Stryker, Kalamazoo, MA, USA) was deployed at the obstructed part of the left MCA through a Trevo Track 21 microcatheter (Stryker, Kalamazoo, MA, USA). Immediately after angiography, recanalization of the MCA (Figure 2B) and the thrombolysis in cerebral infarction (TICI) grades were 3. The time from the last known well to puncture was 310 min, and the time from puncture to recanalization was 62 min. 

**Figure 3:**
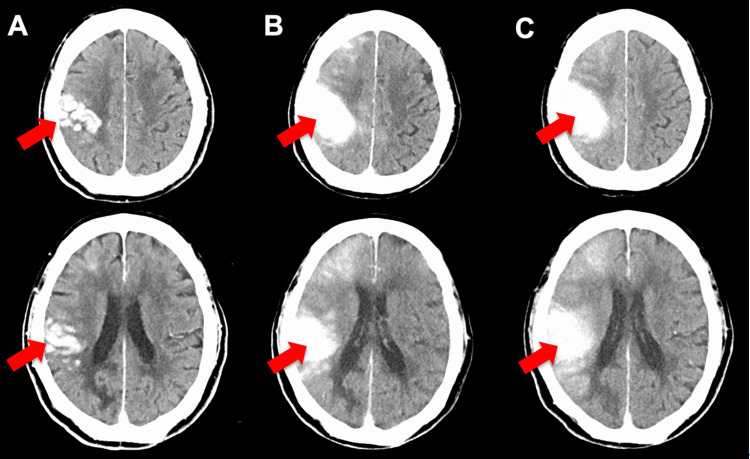
Postoperative progress of head CT A postoperative head computed tomography (CT) scan revealed areas of high density along the cortex in the right frontal and temporal lobes (A: arrow). A head CT scan performed the next day showed that the high-density area had expanded (B: arrow), and the NIHSS score had improved from 23 to 11. A head CT scan taken three days after surgery showed that the high-density area had remained almost completely and was not disappearing (C: arrow). NIHSS: National Institutes of Health Stroke Scale

Postoperative head computed tomography (CT) scan revealed high-density areas along the cortex in the right frontal and temporal lobes (Figure 3A). There was no mass effect, and the CT value was 90 Hounsfield number (HU), and these findings were suggestive of contrast leakage rather than bleeding. A head CT scan performed the following day showed that the high-density area had expanded (Figure 3B), and the NIHSS score had improved from 23 to 11. A head CT scan taken three days after surgery showed that the high-density area remained almost completely and did not disappear (Figure 3C), which was different from the usual progression of contrast leakage. However, the NIHSS score further improved to six points, making it difficult to consider the high-density area as a cerebral hemorrhage.

**Figure 4:**
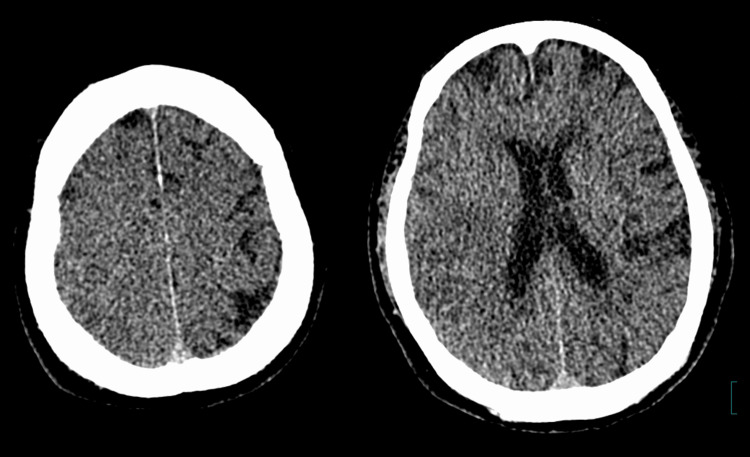
Virtual non-contrast images taken using dual-energy CT In virtual non-contrast images taken using dual-energy CT, the high-density areas seen on plain CT were iso-dense.

Therefore, it was necessary to distinguish between contrast leakage and cerebral hemorrhage. In virtual non-contrast images acquired using dual-energy CT (DECT), these high-density areas were isodense, except for a very small part of the parietal lobe (Figure 4).

**Figure 5:**
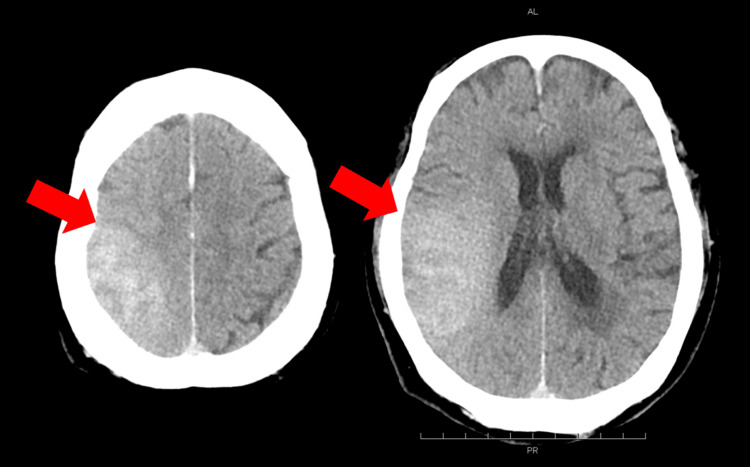
Head CT scan performed immediately after dialysis A CT scan performed immediately after dialysis confirmed the disappearance of the high-density areas (arrow).

Similarly, MRI susceptibility weighted imaging (SWI) revealed no findings suggestive of hemorrhage, apart from a low-signal area in part of the parietal lobe. Based on these findings, the high-density areas on plain CT were determined to be contrast leakage rather than cerebral hemorrhage and were thought to remain for a long period after MT due to delayed excretion of contrast due to renal failure. Therefore, dialysis was initiated five days after MT, and a CT scan performed immediately after dialysis confirmed the disappearance of the high-density areas (Figure 5).

After testing for the cause of the stroke, the patient was diagnosed with cardiogenic cerebral embolism due to severe heart failure and low cardiac function. Warfarin was administered to prevent recurrence. The patient underwent rehabilitation and treatment for heart failure and was discharged to a nursing home approximately 60 days after admission with a modified Rankin scale score of 3.

## Discussion

In recent years, MT has been shown to be highly effective for acute ischemic stroke, and its indications have expanded. As a complication of MT, it is necessary to pay attention to postoperative cerebral hemorrhage, and evaluation using head CT is essential. The hyperdense area on head CT after MT is found in 31.2%~87.5% of cases [1]. These hyperdense areas on non-contrast CT may represent either contrast extravasation or intracranial hemorrhage. 

It is important to distinguish between contrast extravasation and hemorrhage in the immediate postprocedural period because if the cause is hemorrhage, the initiation of antithrombotic therapy must be delayed, but if the cause is contrast leakage, antithrombotic therapy can be administered earlier. This is because re-occlusion occurs in 2.3-7.2% of cases early after MT [3-5], and antithrombotic therapy should be initiated as early as possible to prevent restenosis.

However, distinguishing between contrast extravasation and hemorrhage is difficult. When ischemic injury is limited to the endothelial cell permeability barrier, hyperdensities may represent contrast medium extravasation with no hemorrhagic products, whereas when ischemic injury degrades the basal lamina, hyperdense lesions may be associated with some degree of hemorrhage [6]. 

Definitions and diagnostic criteria for contrast medium leakage and cerebral hemorrhage have not been established and vary in the literature. Mericle et al. reported that contrast extravasation could be defined as a hyperdensity with a maximal Hounsfield unit (HU) measurement > 90 and/or disappearance of the hyperdensity on a repeat CT taken within 24 hours [2]. Nakano et al. reported that hyperdense areas that disappeared the next day were considered contrast-medium extravasation [7]. Yoon et al. reported that most hyperdense lesions that disappeared the next day were due to the contrast agent, and conversely, hyperdense lesions with a maximum HU >90 that remained even on follow-up upgrade CT were associated with parenchymal hematoma [8]. 

As shown above, in previous studies, hyperdense lesions that disappeared early were often diagnosed as contrast agents, while those that persisted for more than 24 hours were often diagnosed as hematomas. However, in this case, the hyperdense lesion caused by contrast medium extravasation expanded in the immediate 24 hours and persisted for five days until dialysis was performed. 

In this case, we finally diagnosed the high-absorption area as contrast agent leakage based on DECT findings and the fact that it was removed after dialysis.

DECT uses two different energy spectra produced by two different energy level settings, either by two X-ray sources or other techniques, and separates the spectra of different materials with different attenuation properties when exposed to different X-ray energies [9]. The usefulness of DECT in differentiating between contrast medium extravasation and hematoma in hyperdense lesions after MT has been reported [10-12]. In this case, DECT was effective in diagnosing contrast leakage.

Contrast media are primarily excreted through glomerular filtration. Therefore, there is a significant correlation between total body clearance of contrast agents and renal clearance and glomerular filtration rate, so renal excretion of contrast agents is delayed in patients with renal insufficiency [13]. In this case, delayed excretion of the contrast agent due to renal failure was thought to be the reason contrast agent leakage continued to be observed on the head CT scan even 24 hours after MT. Therefore, dialysis was performed early to remove the contrast agent, and because it was not a hemorrhagic lesion, antithrombotic therapy for cerebral infarction was started early.

We did not find any previous reports of delayed contrast medium excretion after thrombectomy, as in the present case. The method used in previous studies to distinguish between contrast medium extravasation and hematoma, based on whether the high-density area disappears within 24 hours after MT, may not be applicable to patients with renal failure, as in this case. Therefore, it is necessary to make a comprehensive diagnosis, including image evaluation such as DECT.

## Conclusions

In patients with renal failure, contrast leakage may persist for more than 24 hours after MT owing to the delayed excretion of the contrast agent. Therefore, it becomes difficult to distinguish from bleeding. So, high-density areas on CT after MT in patients with renal failure require detailed examinations, including DECT. In patients with renal failure who have delayed excretion of contrast media, removal of the contrast media by dialysis is effective.
